# Bridging minds: Participant perspectives on postmortem brain research and engagement

**DOI:** 10.1111/neup.13030

**Published:** 2025-02-07

**Authors:** Yusuke Inoue, Maki Obata, Maho Morishima, Shigeo Murayama, Yuko Saito

**Affiliations:** ^1^ Department of Public Policy The Institute of Medical Science, The University of Tokyo Tokyo Japan; ^2^ Department of Healthcare Ethics Kyoto University School of Public Health Kyoto Japan; ^3^ Department of Neuropathology (Brain Bank for Aging Research) Tokyo Metropolitan Geriatric Hospital and Institute of Gerontology Tokyo Japan

**Keywords:** brain banking, ethics, informed consent, patient participation, tissue donors

## Abstract

Postmortem research participation remains underrepresented in research ethics discussions. Herein, we examined the associated perspectives of individuals preregistered with the Brain Bank for Aging Research at the Tokyo Metropolitan Institute of Gerontology. We conducted a postal survey targeting 88 preregistrants, yielding 52 responses (response rate: 59.1%, average respondent age: 79.5 years, range: 49–97). The questionnaire gathered information on the reasons for agreeing to participate, helpful information provided during the explanation, and expectations regarding future information. The stated reasons for participating included a desire to contribute to science, gratitude for medical care received, memories of relatives' past donations, and inspiration from staff enthusiasm and materials. Beneficial information was given in brochures, coordinator explanations, and lectures; however, guidance for family members regarding postmortem procedures and updates on recent activities and research outcomes were highlighted as areas requiring future improvement. Willingness to participate in brain banks was influenced by altruistic factors, personal medical experiences, and the influence of statements from close contacts. Registrants maintained their interest after registration and prepare for future arrangements. Family cooperation was identified as a critical factor influencing the fulfillment of participant intentions, emphasizing the need for accessible and low‐burden family guidance. Registrants generally seek information to help family members and associates avoid difficulties related to their participation. Brain banks should continue conducting such surveys for registrants and reflect the findings in their information dissemination and educational programs. This approach will help improve the understanding and support for brain bank participation, ultimately contributing to the advancement of medical research and ethics in postmortem studies.

## INTRODUCTION

Brain banks that collect brain tissue for research on psychiatric and neurological disorders have been set up in various countries.^1^ In Japan, in addition to individual institutional efforts, a network known as the Japan Brain Bank Net (JBBN) was established to coordinate such initiatives.^2^ However, scandals involving the use of deceased individuals, including brain research, have been reported in several developed countries, undermining public trust in research and resulting in decreased participation.^3^, ^4^ Despite such scandals, ethical considerations regarding “postmortem research participation” continue to lag far behind other areas of medical research, such as the protection of clinical trial subjects. For example, the World Medical Association's Declaration of Helsinki^5^ and the Declaration of Taipei^6^ do not mention postmortem research participation, while the guidelines of the Council for International Organizations of Medical Sciences only briefly state that the principles for protecting living subjects should also be applied to research involving the deceased.^7^

In 2006, the European ethical guidelines on using human tissue for research indicated that procedures should follow consent or “authorization” in accordance with national laws.^8^ These guidelines were revised in 2016 to emphasize that individuals must be properly informed at an earlier time and provided with the option to refuse donations.^9^ While decision‐makers for postmortem donations differ across countries, in some nations, the expectation is that such decisions may be made by the donors themselves, rather than solely by their families.^10^

In Japan, attitudes regarding postmortem tissue donation are deeply influenced by traditional cultural views of death and the human body,^11^, ^12^ which emphasize profound respect for the deceased and their remains, influencing both individual and family decisions about tissue donation. Public trust in postmortem research can be particularly vulnerable to ethical breaches, as demonstrated by a 2024 incident in Japan in which the inappropriate sharing of postmortem images on social media led to significant public concern regarding the handling of human remains.^13^ Such incidents highlight the ongoing need for careful consideration of cultural values and ethical standards in brain banking practices.

This study examined the understanding and perceptions of individuals who decided to donate their postmortem brains to “brain banks” in Japan, where family consent has traditionally placed significant importance on postmortem body donation, as exemplified by the debate regarding brain‐dead organ transplantation. This trend is expected to persist; however, even in such contexts, it is crucial to provide individuals with prior information and to confirm their intention to participate in brain banks. One reason for this is that, despite using an individual's body for research, there has been little emphasis on reflecting the individual's true intentions. This situation is somewhat analogous to the “Advanced Research Directive” currently being trialed in some dementia research, in which individuals express their wishes for situations in which they can no longer communicate their intentions.^14^ Additionally, the lack of clear consent from individuals may complicate decision‐making for families. Discussions regarding postmortem organ and tissue donation indicate that articulated wishes from individuals can significantly influence families' decision‐making.^15^, ^16^

Some brain banks within the JBBN have implemented initiatives that request that individuals indicate their intentions as an option. This is referred to as “expression of intention during lifetime” or “lifetime registration” and has been mentioned in the guidelines of relevant academic societies in Japan.^17^ However, no examination yet exists on how individuals react to explanations and information provided when confirming donation intentions during their lifetime. Consequently, this study conducted interviews with those who had completed lifetime registration, aiming to gain insights into future explanation content.

## MATERIALS AND METHODS

### Participants and selection criteria

This study targeted individuals who had been preregistered with the Brain Bank for Aging Research at the Tokyo Metropolitan Institute for Geriatrics and Gerontology (TMIGG) and agreed to respond to the survey (preregistrants). Individuals with impaired decision‐making capacities and their families were excluded from the study. While this study assumed that participants would respond by themselves, the possibility of close relatives submitting responses on their behalf was acknowledged.

### Recruitment method and study period

Data were collected through a questionnaire survey administered by mail. The survey forms were sent on 8 May 2023, with a response deadline of 30 June 2023. The questionnaire items described below were sent to individuals preregistered with the brain bank. The mailing utilized registration information held by the TMIGG.

### Survey items

The survey aimed to gather opinions on the brain bank's public relations and information dissemination, as well as feedback on and evaluations of current explanatory materials. The questionnaire included questions obtaining information on the following:

1. Demographic information (sex, age, education level, place of residence).
2. Reasons for donor registration.
3. Information deemed useful when considering participation in the brain bank.
4. Information desired from the brain bank in the future.

Questions 2–4 were multiple‐choice but included an “Other” option to allow for additional reasons, for which free‐text fields were provided for the participants to freely express their thoughts.

### Quantitative methods

Fisher's exact test (two‐sided) was applied for between‐group comparisons. All statistical analyses were conducted using IBM spss Statistics version 25 (IBM Corp., Armonk, NY, USA). Statistical significance was set at *P* < 0.05.

### Qualitative methods

The free‐text responses were analyzed using thematic analysis. Two researchers independently coded the responses and identified emerging themes. Disagreements were resolved through discussion with a third researcher. The final themes were reviewed by all authors to ensure their validity and relevance to the research questions.

### Disclosure of ethical statements

This study was approved by the Institute of Medical Science, University of Tokyo (approval number: 2022‐73‐0228, February 2022) and the Tokyo Metropolitan Institute of Gerontology (approval number: R23‐002, May 2023).

Potentially identifying information, such as specific institution names, disease names, names of doctors or staff, and temporal information, was removed from all free‐response sections and were replaced with mechanically generated random alphabetical expressions (Supplements 1–3 in Data S1).

## RESULTS

### Result 1: Demographic characteristics

From the 88 surveys sent, 52 responses were received (response rate: 59.1%). Respondents included 23 males and 29 females, with an average age of 79.5 years (range: 49–97 years). Forty‐one participants responded personally, while 11 responses were submitted by proxies (Table 1).

**Table 1:** Participant demographics (*N* = 52)

Characteristic	Category	*n*	Percentage (%)
Sex	Male	23	44.2
	Female	29	55.8
Age	≤50s	3	5.8
	60s	6	11.5
	70s	14	26.9
	≥80s	29	55.8
Education	University	21	40.4
	Junior college/vocational school	8	15.4
	High school	17	32.7
	Junior high school	3	5.8
	Other	3	5.8
Residence	Kanto region	49	94.2
	(Tokyo)	(37)	(71.2)
	(Other Kanto)	(12)	(23.1)
	Other regions	3	5.8
Respondent	Self	41	78.8
	Proxy	11	21.2

Interestingly, a relationship between age and gender distribution was found. While the gender ratio showed no substantial difference in individuals in their 70s (six males, eight females) and 80s (16 males, 13 females), females accounted for 88.9% of those aged 60 and younger (one male, eight females). As subsequently discussed, these relatively younger individuals were predominantly female caregivers accompanying patients, rather than the patients themselves.

### Result 2: Reasons for registration

All 52 respondents answered the multiple‐choice question regarding their reasons for registering with the brain bank, with 13 providing additional comments in the free‐text field (Tables 2 and 3 and Supplement 1 in Data S1). Many respondents provided several reasons for their decision to register with the brain bank. Among the predefined options, “contribute” (desire to contribute) and “useful” (desire to be useful) were chosen most frequently (Table 2). The free‐text responses are summarized in Tables 4 and 5. While many responses overlapped with the multiple‐choice options, they also revealed gratitude for medical care received by respondents or their family members as a significant motivating factor. Additionally, staff explanations and informational materials were found to play an important role in the decision to register.

**Table 2:** Responses regarding brain bank registration background and useful information (*N* = 52)

Question	Response option	*n*	Percentage (%)
Reasons for donor registration (multiple answers allowed)	To contribute to disease elucidation and future medical care	45	86.5
	To be useful even after death	43	82.7
	Interest in social contribution	25	48.1
	Have (had) someone close with a brain‐related illness (including the registrant)	21	40.4
	To receive a final pathological diagnosis	11	21.2
	Recommended by an attending physician	6	11.5
	Other	8	15.4
Information useful when considering brain bank participation (multiple answers allowed, four non‐responses)	Brain bank brochures, guidebooks, and other informational materials	34	70.8
	Explanation from the brain bank office (coordinators and others)	30	62.5
	Explanation from attending physician or doctors	17	35.4
	Content from lectures and so forth	14	29.2
	The brain bank (Tokyo Metropolitan Institute of Gerontology) website	10	20.8
	Other	7	14.6
Beneficial items from brain bank mailings (multiple answers allowed, eight non‐responses)	The brain bank newsletter	31	70.5
	Revised “Explanatory Materials for Family Members” from the brain bank	28	63.6
	Reference materials (health‐related, such as “dementia,” “rehabilitation,” “nutrition and diet,” “oral care,” and “other”)	28	63.6
	Brain bank lecture announcements	22	50.0
	Other	1	2.3

**Table 3:** Responses regarding brain bank registration background, classified by age (in decades) and sex

Question	Response option	≤60s (*n* = 9)	70s (*n* = 14)	80s≥ (*n* = 29)	Male (*n* = 23)	Female (*n* = 29)
		*n* (%)	*n* (%)	*n* (%)	*n* (%)	*n* (%)
Reasons for donor registration (multiple answers allowed)	To contribute to disease elucidation and future medical care	6 (66.7)	13 (92.9)	26 (89.7)	19 (82.6)	26 (89.7)
	To be useful even after death	6 (66.7)	13 (92.9)	24 (82.8)	18 (78.3)	25 (86.2)
	Interest in social contribution	2 (22.2)	10 (71.4)	13 (44.8)	10 (43.5)	15 (51.7)
	Have (had) someone close with a brain‐related illness (including the registrant)	5 (55.6)	5 (35.7)	11 (37.9)	5 (21.7)*	16 (55.2)*
	To receive a final pathological diagnosis	2 (22.2)	3 (21.4)	6 (20.7)	5 (21.7)	6 (20.7)
	Recommended by an attending physician	1 (11.1)	3 (21.4)	2 (6.9)	4 (17.4)	2 (6.9)
	Other	2 (22.2)	1 (7.1)	5 (17.2)	5 (21.7)	3 (10.3)

* Significant difference between males and females (*P* < 0.05 by Fisher's exact test).

**Table 4:** Summary of free‐form responses regarding reasons for participating in a brain bank

Perspective	Examples of responses provided in free‐form entries. For response numbers, refer to Supplement 1 in Data S1
Gratitude toward medical care and institutions	“Since falling ill, I have received care from many medical institutions. I believe these treatments have incurred significant expenses for others as well; consequently, I decided to contribute in hopes of also providing some assistance.” (a) “When I registered as a donor, my spouse was healthy, and I never imagined they would develop [Condition A] and require care. Looking back, I now wonder if I could be of some help.” (b) “I live in [Prefecture X] but receive medical care in [Prefecture Y], so I thought I could contribute in some way.” (e)
Desire to contribute to disease research and be useful	“As their sibling, I may be useful for genetic comparison.” (f) “When my parent's brain was studied by [Doctor A] after suffering from [Condition D], I learned about the tissue bank and the need for donors. I thought having both parent and child samples may be beneficial for research, including in genetic studies.” (k)
Influence of family members and surroundings	“Because I, the spouse, registered first and encouraged my partner to follow.” (c) “My spouse used to work at [Hospital Z], where they performed medical research. That's why I supported the establishment of the tissue bank and registered as a donor.” (g) “A family member is a doctor. I am turning [Age X] and am suddenly becoming aware of my decline. I hope my registration can be of use.” (j) “I am the only one in my family to reach this age. I have always thought this might be because my cognitive functions are slower than most. I wonder if studying my brain could shed light on this.” (l) “Around [Year X], a family member was diagnosed with [Condition F] at [Hospital G]. The way it was communicated was very shocking. Later, I attended [Event A] on tissue donation and learned about the process.” (m)
Influence of coordinator's enthusiasm and brain bank pamphlet information	“I became interested after reading about the tissue bank's activities in a pamphlet.” (i) “I was moved by the explanation given by [Person A] (the coordinator).” (d)

**Table 5:** Summary of requests for information from brain bank

Perspective	Examples of responses provided in free text (response numbers refer to Supplement 3 in Data S1)
Knowledge about one's own body or treatment (seeking information about questions related to one's own condition or consultation resources)	“I would like to receive a diagnosis at [Institution A] and live healthily. Please let me know about the contact point for consultations.” (a)
	“I would like to receive new information for reference, if available.” (e)
	“I would like to see the lectures held again, as they were canceled due to COVID‐19.” (h)
	“Information on daily health practices individuals can adopt to prevent cognitive decline.” (i)
Understanding and questions about research participation	“I have been receiving medication and follow‐up care from neurosurgery for [Condition A], which began in 2014. I am wondering if progress reports on this are necessary.” (c)
	“I have had a stressful life since childhood. I would like research to be done on the brains of people like me as well.” (l)
Actions to be taken by the bereaved family and surrounding individuals	“My family has agreed, but I want to know about the actual procedure. I would like to document the contact information, relationship with the hospital, and the number of days until my body is returned.” (b)
	“I plan to write my will in June or July at a notary office. Since strangers will handle my postmortem affairs, I would appreciate any relevant precautions.” (l)
	“Easy‐to‐understand materials or brochures about the appropriate process after death (the after death certificate).” (m)
	“I would like to know specifically how the autopsy results are being used. As a donor, I would also like to know how brains are disposed of after they have served their purpose in research.” (p)
Recent status of the brain bank and means to know about it	“Statistical data such as the number of donors and age group ratios.” (j)
	“I would appreciate a summary of the lectures after they conclude.” (k)
	“When I asked a classmate about the brain bank, they hardly knew anything about it. I wish more people from middle to old age would cooperate (my classmate is in Hokkaido).” (o)
	“As I have become a caregiver (at home), I have not been able to attend lectures or review materials. I only see posters at the hospital. Are lectures still being held? I would like to know the current number of brain donation registrants, unless I have overlooked this information.” (t)
Examples of research use and research progress	“I would like to know specific examples of how the research results are being utilized.” (n)
	“The results of research to date.” (q)
	“Although it is becoming difficult for me to attend lectures, I would like information or materials about specific examples of how donations are actually being used in research.” (s)
	“A straightforward summary of the research progress easy for laypersons to understand, along with an introduction to international research systems and approaches.” (u)
No particular request/other	“I do not have any particular requests at the moment.” (d)
	“I think it would be good if everyone in Japan could have their cause of death determined and if all usable organs could be utilized. I would appreciate information on why this is not possible yet and how it is progressing.” (f)
	“I am interested in updates regarding major revisions to the brain bank's operations. I registered based on my own beliefs, so I do not particularly need other information.” (g) “I do not have a computer or smartphone, so I am not good with IT at all. Also, I tend to avoid difficult things even in writing.” (r)

Gender analysis revealed that medical contribution and postmortem experience were the most impactful factors for both groups (Table 3). However, a notable gender disparity was identified in the “influence of close relations” category, with female participants showing a significantly higher selection rate. Age‐based analysis revealed no statistically significant differences, although certain distinctive patterns were observed. Specifically, respondents in their 70s and 80s predominantly selected “contribution” factors (social contribution and postmortem contribution), while participants under 60 years of age were more likely to report that the “influence of close relations” was a major factor in their participation.

These combined results appeared to be influenced by gender demographics. As noted in Result 1, our analysis revealed a notable female predominance, particularly among younger age groups. The higher proportion of younger female registrants may be attributed to their involvement as caregivers accompanying elderly patients for treatment. This indicates that exposure to the program through accompanying relatives to their medical treatment serves as a crucial entry point for participation in this activity.

### Result 3: Information perceived as beneficial

Overall, 48 respondents answered the question regarding useful information sources when registering (Table 2). The most frequently cited sources were explanatory materials, including the brochure and other materials created by the institution.^18^ This was followed by “explanations from coordinators and others,” “explanations from attending professionals/doctors,” and “information from lectures.” Similarly, 44 respondents provided more detailed answers regarding the most beneficial information materials (Table 2). While half identified lectures as beneficial, the most frequently mentioned resource was tangible materials, including newsletters that can be retained for reference.

These responses highlight the importance of the materials and support provided by the administrative offices. Indeed, five free‐text responses mentioned previous experiences with family donations, trust in doctors and coordinators, and telephone support (Supplement 2 in Data S1).

### Result 4: Desired information and guidance

A total of 21 respondents provided feedback on the information they would like to receive in the future (Supplement 3 in Data S1), which could be broadly categorized into the following categories: “knowledge about one's own body and treatment,” “questions about research participation,” “actions to be taken by bereaved family and surrounding individuals,” “recent activities of the brain bank,” and “examples of research use and progress.” Additionally, two responses indicated no specific requests.

Responses regarding “actions to be taken by bereaved family and surrounding individuals” indicated that registrants were concerned about the situations their survivors would face after their death. There were several notable requests for information regarding the appropriate processes to be followed after death as well as the procedures to be carried out by family members and associates at the time of death. Specifically, registrants deemed it necessary to provide information on how family members and others in their surroundings should respond and engage after death (Table 5).

Registrants further showed a high level of interest in the bank's operational status. Beyond the brain bank's activities, such as the number of registrants, a significant interest was shown in how the donated brains would be utilized, including autopsy results, research content, and research outcomes. Furthermore, some responses indicated an interest in receiving the latest updates regarding the brain bank's activities and its overall direction.

## DISCUSSION

Overall, discussions on postmortem research participation are underrepresented in research ethics debates. In this study, we surveyed individuals preregistered with the brain bank to understand their expectations and perceptions concerning the brain bank's public relations, information dissemination, and current explanatory materials. These findings will enhance the understanding and support for brain bank participation, ultimately advancing medical research and ethics in postmortem studies.

The intentions expressed by these individuals were shaped by their accumulated life experiences (Result 2). As presented in our analysis of the participants’ reasons for registering, most participants (86.5%) expressed a desire to contribute to disease elucidation and future medical care. Among younger participants, the presence of family members affected by relevant diseases served as a significant motivating factor. The free‐text responses further revealed additional motivations, including gratitude toward medical professionals and the healthcare system itself, as well as insights influenced by their relationships with those around them. Of particular note, many participants viewed their donation not merely as a provision of tissue but as a meaningful contribution shaped by personal experiences, gratitude toward medical care, and hope for future medical advancement. Understanding these complex motivations is crucial for developing appropriate approaches to donor recruitment and retention.

It is important that these motivations be considered within the broader cultural context of Japanese society, particularly in regard to postmortem tissue donation. Japanese society traditionally holds strong views about the dignity of human remains and the importance of proper treatment of the deceased.^11^ These cultural values significantly influence both individual decisions regarding tissue donation and family responses to such decisions. Public sensitivity to these issues was notably demonstrated in late 2024, when the inappropriate sharing of postmortem images on social media by a medical professional sparked widespread public discourse regarding ethical standards in handling human remains.^13^ Although this incident was not directly related to research activities, it reinforced the critical importance of maintaining rigorous ethical standards to ensure high public trust in all aspects of postmortem tissue handling.

Given these cultural considerations and participant motivations, our findings indicate several practical implications for brain bank operations and communication strategies. While a discussion of the artificial means of creating initial contact points by researchers is beyond the scope of this paper, our survey findings provide several insights about how to support the actions and communication of those interested in brain banking. To effectively communicate about brain banks, it is essential to prepare clear explanations regarding the concept itself. Overall, our findings demonstrate that participants valued tangible materials in particular, with 70.8% finding brain bank brochures and guidebooks helpful when considering participation. Furthermore, 62.5% of participants highlighted the importance of explanations from brain bank coordinators; as such, the presence and quality of the personnel supporting the explanation are crucial.

Coordinators serve as a vital link between brain banks and society, significantly reducing the psychological distance between individuals and the activities of the brain bank. Furthermore, participants expressed strong interest in receiving updates regarding research progress and outcomes, indicating their desire to remain connected to the program even after registration. This ongoing engagement could help to maintain public trust and support for postmortem tissue donation programs.

The emphasis participants placed on family involvement and support was another significant finding of our analysis. Participants who expressed a willingness to participate recognized that they could not fulfill their research participation alone. Multiple respondents specifically requested information regarding postmortem procedures and guidance for family members, indicating their concerns about minimizing burden on their families. These results agree with those of previous studies on postmortem donations, in which family support was identified as a critical factor influencing successful donation programs. It is important that guidance for families be appropriately presented and that such guidance be shown in an understandable and low‐burden form; indeed, a brochure from the UK Parkinson's Disease Brain Bank provides similar information.^19^ As brain banks shift from “a surrogate consent scheme (by the bereaved family)” to obtaining consent directly from individuals, the importance of family cooperation and clear guidance is becoming increasingly significant.

Several limitations should be considered regarding this survey targeting a brain bank for older adults. For example, respondents may have favored the medical institution's activities or prioritized maintaining a good relationship with the institution in their responses. Moreover, the small sample size of this study may not have allowed adequate representation of the diverse interests and concerns of the population. However, because this study specifically aimed to inquire about participants' reasons for participation and their desires at the time, the questions were less likely to be influenced by conflicts of interest with the institution. Furthermore, considering the scarcity of similar reports, these testimonies are valuable. Additionally, a notable characteristic of this study is that the respondents, primarily older adults, represent a group that has given considerable thought to postmortem research participation, viewing it as a pressing issue.

While our findings provide valuable insights, several important questions remain for future research. For example, there is a need to better understand the relationship between registrants and their families, including how registrants overcome any discrepancies between their perceptions and those of their families. Indeed, family cooperation is essential for donating body parts after death. However, there is a possibility of discrepancy between individual and family intentions regarding postmortem organ donation.^20^ Thus, it is important to understand how individuals communicate with their families when expressing their intentions, both during and after the discussion. In the future, interviews with registrants regarding these topics will be essential.

It is important to note that this study primarily involved older adults; as such, the expression of willingness to donate reflects the culmination of a patient's life experiences. Many respondents made well‐considered declarations of intent, indicating a broad range of applications when considering how to engage the public and facilitate decision‐making in the future. However, separate considerations may be necessary depending on the characteristics of the disease and the influence of age. For example, there is a possibility that gratitude toward medical care and medical professionals is more prominent in older adults. However, few people are unaffected by medical care, and we believe that the basic policy remains unchanged, even when calling for the donation of healthy brains.

In conclusion, this study found that ensuring opportunities to listen to registrants' stories and concerns is an essential aspect of patient and public involvement in brain banks. The results obtained are likely to include lessons that could inform the development of educational activities and materials for researchers and medical professionals. Further, our findings highlight the complex interplay between personal motivations, cultural values, and family dynamics in postmortem tissue donation. While challenges remain, particularly regarding family communication and age‐specific considerations, this study nevertheless provides a foundation for improving brain bank practices and public engagement strategies. Our research team has already begun further investigations into these areas and will continue to explore these important aspects of brain bank participation.

## DISCLOSURE

The authors declare no conflicts of interest related to this article.

## Supporting information


**Data S1** Supporting Information.

## Data Availability

The data that support the findings of this study are available on request from the corresponding author. The data are not publicly available due to privacy or ethical restrictions.
