# Supplementary material for: Bridging minds: Participant perspectives on postmortem brain research and engagement
Source: Neuropathology. 2025 Feb 7;45(4):e13030. doi: 10.1111/neup.13030 (PMC12279472; doi:10.1111/neup.13030)
Supplement: Supplementary file 1 — Data S1 Supporting Information. [file NEUP-45-0-s001.docx]

**Supplement 1: Free-text Responses (Reasons for Registering with the Brain Bank)**

| a. Since falling ill, I have received care from various medical institutions, which I believe has incurred significant expenses for others. Consequently, I decided to contribute in hopes of providing some assistance. It would be even more helpful if［Institution A］ could check on my progress afterward.  b. When I registered as a donor, my spouse was healthy, and I never imagined they would develop [Condition A] and need care. Looking back, I now wonder if I can be of help. Currently, my spouse has been diagnosed with ［Condition B］ and ［Condition C］. We are scheduled for a meeting about treatment options. Despite these conditions, they are not in pain and still have an appetite. We have been receiving care from ［Hospital X］, ［Hospital Y］, and various other facilities.  c. Because I, the spouse, registered first and encouraged my partner to follow.  d. I was moved by the explanation given by ［Person A］ (the coordinator).  e. I live in ［Prefecture X］ but receive medical care in ［Prefecture Y］, so I thought I could contribute in some way.  f. As their sibling, I may be useful for genetic comparison.  g. My spouse used to work at ［Hospital Z］, where they performed medical research. I supported the establishment of the tissue bank and registered as a donor. The hospital's name has changed since then.  h. The individual has little interest in such initiatives due to their condition and maintains a neutral stance. We registered as donors based on the family's wishes, and he agreed to participate.  i. I became interested after being informed about the tissue bank’s activities in a pamphlet.  j. A family member is a doctor. I am turning ［Age X］ and suddenly aware of my decline. I hope my registration can be of use.  k. When my parent's brain was studied by ［Doctor A］ after suffering from ［Condition D］, I learned about the tissue bank and the need for donors. I thought having both parent and child samples might be beneficial for research including genetic studies. I registered hoping for this. I was recently diagnosed with ［Condition E］. If you need any clinical data from me for future research, please let me know.  l. I am the only one in my family to reach this age. I have always thought this might be because my cognitive functions are slower than most. I wonder if studying my brain could shed light on this.  m. Around ［Year X］, a family member was diagnosed with ［Condition F］ at ［Hospital G］. The way it was communicated was very shocking. Later, I attended ［Event A］ on tissue donation and learned about the process. I registered, and when my family member passed away, we donated their tissue. I am glad if it can contribute to research. |
| --- |

## **Supplement 2: Free-text Responses (Information Found Beneficial When Registering with the Brain Bank)**

| a. It's “trust” in ［Doctor A］ and ［Person B］ (the coordinator).  b. (Regarding the revised "Explanation Materials for Families" of the brain bank) What's that?  c. Although it was not through mail, the coordinator I spoke with several times on the phone provided a very helpful response.  d. I cannot often attend the lectures, but I always want to go.  e. The response of ［Doctor C］ who was in charge during the brain donation, and the talk by ［Doctor D］ at the lecture - both doctors left a deep impression on me. |
| --- |

## **Supplement 3: Free-text Responses (Information Expected from the Brain Bank)**

| a. I would like to receive a diagnosis at ［Institution A］ and live healthily. Please let me know about the contact point for consultations.  b. My family has agreed, but I want to know about the actual procedure. I would like to document the contact information, relationship with the hospital, and the number of days until the body is returned.  c. I have been receiving medication and follow-up care from neurosurgery for ［Condition A］, which began in 2014. I am wondering if progress reports on this are necessary.  d. I don't have any particular requests at the moment.  e. I would like to receive new information for reference if available.  f. I think it would be good if everyone in Japan could have their cause of death determined, and if all usable organs could be utilized. I would appreciate information on why this is not possible yet and how it is progressing.  g. I am interested in updates regarding major revisions to the brain bank's operations. I registered based on my own beliefs, so I do not particularly need other information.  h. I would like to see the lectures held again, as they were canceled due to COVID-19.  i. Information on daily health practices individuals can adopt to prevent cognitive decline.  j. Statistical data such as the number of donors and age group ratios.  k. I would appreciate a summary of the lectures after they conclude.  l. I plan to write my will in June or July at a notary office. Since strangers will handle my post-mortem affairs, I would appreciate any relevant precautions. I have experienced a stressful life since childhood and would like research conducted on the brains of individuals like me as well.  m. Easy-to-understand materials or brochures about the appropriate process after death (after death certificate).  n. I would like to know specific examples of how the research results are being utilized.  o. When I asked a classmate about the brain bank, they hardly knew anything about it. I wish more people from middle age to old age would cooperate (my classmate is in Hokkaido).  p. I would like to know specifically how the autopsy results are being used. As a donor, I would also like to know how brains are disposed of after they have served their purpose in research.  q. The results of research to date.  r. I turned 90 last November. I do not have a computer or smartphone, so I am not good with IT at all. Also, I tend to avoid difficult things even in writing.  s. Although it is becoming difficult for me to attend lectures, I would like information or materials about specific examples of how donations are actually being used in research.  t. As I have become a caregiver (at home), I have not been able to attend lectures or review materials. I only see posters at the hospital. Are lectures still being held? I would like to know the current number of brain donation registrants unless I have overlooked this information.  u. A straightforward summary of the research progress that is easy for laypersons to understand, along with an introduction to international research systems and approaches. |
| --- |
